# Update on Lysinuric Protein Intolerance, a Multi-faceted Disease Retrospective cohort analysis from birth to adulthood

**DOI:** 10.1186/s13023-016-0550-8

**Published:** 2017-01-05

**Authors:** Wladimir Mauhin, Florence Habarou, Stéphanie Gobin, Aude Servais, Anaïs Brassier, Coraline Grisel, Célina Roda, Graziella Pinto, Despina Moshous, Fahd Ghalim, Pauline Krug, Nelly Deltour, Clément Pontoizeau, Sandrine Dubois, Murielle Assoun, Louise Galmiche, Jean-Paul Bonnefont, Chris Ottolenghi, Jacques de Blic, Jean-Baptiste Arnoux, Pascale de Lonlay

**Affiliations:** 1 Reference Center of Inherited Metabolic Diseases, Imagine Institute, Hospital Necker Enfants Malades, APHP, University Paris Descartes, Paris, France; 2 Metabolic Biochemistry, Hospital Necker Enfants Malades, APHP, University Paris Descartes, Paris, France; 3 Molecular Genetics, Hospital Necker Enfants Malades, APHP, University Paris Descartes, Paris, France; 4 Nephrology Unit, Hospital Necker Enfants Malades, APHP, University Paris Descartes, Paris, France; 5 Endocrinoloy Unit, Hospital Necker Enfants Malades, APHP, University Paris Descartes, Paris, France; 6 Paediatric Immunology, Haematology and Rheumatology, Hospital Necker Enfants Malades, APHP, University Paris Descartes, Paris, France; 7 Pneumology, Hospital Necker Enfants Malades, AP-HP, University Paris Descartes, Paris, France; 8 Gastroenterology, Kremlin Bicêtre Hospital, AP-HP, University Paris Sud, Paris, France; 9 Nephrology, Hospital Necker Enfants Malades, APHP, University Paris Descartes, Paris, France; 10 Anatomopathology, Hospital Necker Enfants Malades, APHP, University Paris Descartes, Paris, France; 11 Reference Center of Metabolic Disease Unit, Université Paris Descartes, Hôpital Necker-Enfants Malades, Institute Imagine, INSERM-U781, 149 rue de Sèvres, 75015 Paris, France

**Keywords:** Lysinuric protein intolerance, Inborn error of metabolism, Hyperammonemia, Urea Cycle disorder, Pulmonary alveolar proteinosis, Myocardial infarction, Hemophagocytic lymphohistiocytosis, Lysine, Lupus, Amyloidosis

## Abstract

**Background:**

Lysinuric protein intolerance (LPI) is a rare metabolic disease resulting from recessive-inherited mutations in the *SLC7A7* gene encoding the cationic amino-acids transporter subunit y^+^LAT1. The disease is characterised by protein-rich food intolerance with secondary urea cycle disorder, but symptoms are heterogeneous ranging from infiltrative lung disease, kidney failure to auto-immune complications. This retrospective study of all cases treated at Necker Hospital (Paris, France) since 1977 describes LPI in both children and adults in order to improve therapeutic management.

**Results:**

Sixteen patients diagnosed with LPI (12 males, 4 females, from 9 families) were followed for a mean of 11.4 years (min-max: 0.4-37.0 years). Presenting signs were failure to thrive (*n* = 9), gastrointestinal disorders (*n* = 2), cytopenia (*n* = 6), hyperammonemia (*n* = 10) with acute encephalopathy (*n* = 4) or developmental disability (*n* = 3), and proteinuria (*n* = 1). During follow-up, 5 patients presented with acute hyperammonemia, and 8 presented with developmental disability. Kidney disease was observed in all patients: tubulopathy (11/11), proteinuria (4/16) and kidney failure (7/16), which was more common in older patients (mean age of onset 17.7 years, standard deviation 5.33 years), with heterogeneous patterns including a lupus nephritis. We noticed a case of myocardial infarction in a 34-year-old adult. Failure to thrive and signs of haemophagocytic-lymphohistiocytosis were almost constant. Recurrent acute pancreatitis occurred in 2 patients. Ten patients developed an early lung disease. Six died at the mean age of 4 years from pulmonary alveolar proteinosis. This pulmonary involvement was significantly associated with death. Age-adjusted plasma lysine concentrations at diagnosis showed a trend toward increased values in patients with a severe disease course and premature death (Wilcoxon *p* = 0.08; logrank, *p* = 0.17). Age at diagnosis was a borderline predictor of overall survival (logrank, *p* = 0.16).

**Conclusions:**

As expected, early pulmonary involvement with alveolar proteinosis is frequent and severe, being associated with an increased risk of death. Kidney disease frequently occurs in older patients. Cardiovascular and pancreatic involvement has expanded the scope of complications. A borderline association between increased levels of plasma lysine and poorer outome is suggested. Greater efforts at prevention are warranted to optimise the long-term management in these patients.

## Background

Lysinuric protein intolerance (LPI; OMIM #222700) is a rare inborn metabolic disease resulting from recessive-inherited mutations involving the *SLC7A7* gene [1, 2]. LPI has been described sporadically worldwide and has a higher prevalence in Finland (1/60 000) [3, 4]. Defects occur in the y^+^LAT1 sub-unit of the cationic amino-acids transporter localized at the basolateral membrane of the tubular kidney and small bowel cells, leading to the classical hallmarks of the disease: leakage of cationic amino-acids in the urine (arginine, ornithine, lysine) with associated normal to low plasma levels. y^+^LAT1 is also expressed in the lung and spleen and in circulating monocytes and macrophages, which would explain the wide spectrum of symptoms that has been described, such as failure to thrive, protein intolerance, hepatosplenomegaly, osteoporosis, lung involvement, kidney failure, immunological disorders with autoimmunity and haemophagocytic-lymphohistiocytosis. Neurological impairment has also been reported due to the secondary urea cycle disorder. The therapeutic management is thus focused on the preventive care of hyperammonemia and nutritional deficiencies. Together, these approaches have led to benefits in terms of life expectancy. Nevertheless, there is a need to improve our management of the disease and to describe and anticipate the problems faced by young adults today. With this retrospective study, we aimed to describe the multi-faceted presentation of LPI and the current therapeutic strategies [3, 4].

## Methods

### Data

**Fig. 1:**
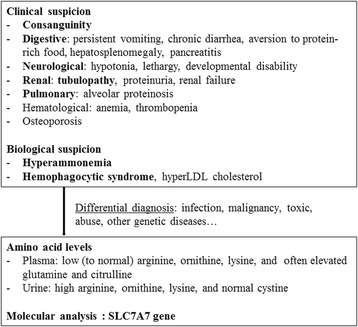
Diagnosis flowchart for LPI

We performed a retrospective analysis of the medical records of all patients identified at Necker Hospital (Paris, France) with a diagnosis of LPI from January 1977 to July 2015. The screening was performed using the CEMARA database for rare diseases and the archives of the Metabolic Biochemistry Department. LPI was suspected on high urinary and low to normal plasma levels of arginine, ornithine and lysine, other biochemical abnormalities, yet normal cystine on amino-acid chromatography and a suggestive clinical presentation (Fig. 1). Molecular investigation was performed to identify mutations in the *SLC7A7* gene. Genomic DNAs were extracted from leukocytes. The nine coding exons and intron-exon boundaries of the *SLC7A7* gene (NM_001126106.2) were amplified by standard polymerase chain reaction (PCR) and analysed by direct sequencing on an ABI3100 automatic sequencer (Applied Biosystems, France). The primers used for PCR and sequencing were designed with the Primer 3 software (http://bioinfo.ut.ee/primer3-0.4.0/primer3/). The potential impact of the non-described variant at the protein level was predicted *in silico* using Polyphen 2 (http://genetics.bwh.harvard.edu/pph2/).

Pulmonary disease was assessed according to the results of broncho-alveolar lavages (BAL), chest X-ray or high-resolution computed tomography (HRCT) and lung biopsy, when available. Renal disease was defined as the presence of tubulopathy, microalbuminuria, proteinuria or estimated glomerular filtration rate (GFR) less than 60 ml/min. GFR was estimated using the Schwartz formula [5] for those under 16 years of age or the Modification of Diet in Renal Disease MDRD equation [6] for older individuals. Renal tubulopathy was defined as glycosuria with concomitant normal glycaemia and/or beta-2-microglobulinuria. Cerebral impairment was defined as developmental disability or hypotonia observed for more than 6 consecutive months. Osteopenia was defined radiologically and/or with a Z-score < -1 standard deviation (SD) on bone densitometry. Haemophagocytic-lymphohistiocytosis was defined according to the criteria outlined by the HLH-2004 [7].

### Statistical analyses

Fisher’s exact test was used to assess the association between qualitative variables. The Mann-Whitney test was performed to compare groups when variables were continuous. The amino-acid levels between living and deceased patients were compared by Wilcoxon test after age adjustment of log-transformed plasma levels at diagnosis. Siblings of index patients were included in the analyses because they had similar outcome (3/9 premature deaths in index cases vs. 2/5 deaths in siblings; see table). Missing data were excluded in the statistical analyses.

Statistical analyses were performed in the R environment (cran.r-project.org/).

### Ethics

This work was approved by our institutional Ethics Committee after declaration to the *Département de la Recherche Clinique et du Développement*, and informed consent was obtained from the parents of underage patients or from adult patients.

## Results (Tables 1 and 2)

**Table 1:** Clinical characteristics of patients with LPI. (Asterisks refer to the same familial pedigree)

Patient	1 *	2 *	3 **	4 **	5 ***	6	7 ***	8 ****	9	10	11	12	13 ****	14 *	15 *	16 ***	n/N or mean (SD)
Year of birth	1976	1977	1977	1980	1991	1993	1994	1994	1996	1997	1997	1999	2001	2003	2005	2010	
Sex	f	m	m	m	m	m	m	f	m	m	m	f	f	m	m	m	m/f : 3
Consanguinity	+	+	-	-	+	-	+	+	+	+	-	+	+	+	+	+	12/16
Age at diagnosis (y.)	1.5	0.8	16.2	13	2.2	1.3	0	0.7	2.2	1.3	12.6	3.4	10.3	0.1	0.005	0.008	4.1 (4.5)
Death	+	-	-	-	-	-	+	+	+	-	-	-	-	+	+	-	6/16
Age at last visit or death (y.)	10.1	37.8	37.4	34.6	23	20.2	5.1	1.8	4.5	18.1	18.1	15.8	13.6	0.5	2.1	5.1	15.5 (10.1)
Failure to thrive	+	+	+	+	+	+	+	+	+	+	+	+	+	+	+	+	16/16
Hepato splenomegaly	+	+	+	+	+	+	+	+	+	+	+	+	+	+	+	+	16/16
Alveolar proteinosis	+	+	-	-	+	+	+	+	+	-	-	-	-	+	+	+	10/16
Renal tubulopathy	+	+	+	+	+	+	NA	NA	NA	+	+	+	+	NA	NA	+	11/11
Proteinuria > 0,3g/l	-	+	+	-	-	-	NA	-	NA	+	+	-	-	NA	NA	-	4/16
eGFR < 90ml/min	-	+	+	+	-	+	-	-	-	+	-	-	+	-	-	-	6/16
Histological associated disease pattern	hepatic amyloidosis	-	-	renal amyloidosis	-	-	-	-	-	-	lupus membranous nephritis	-	Intersitial nephritis - renal fibrosis	-	-	-	
Cognitive impairment	NA	-	+	+	+	-	NA	+	+	+	-	-	-	+	+	-	8/14
Hemophagocytosis	NA	-	+	+	-	+	+	-	+	-	-	-	-	-	-	-	5/15
Dysimmunity	-	-	-	-	+	-	+	-	-	-	+	-	-	-	-	-	3/16
Osteopenia	+	+	-	+	+	+	NA	+	+	+	+	+	+	-	+	-	12/15
Pancreatitis	-	-	-	-	+	-	-	-	-	-	-	+	-	-	-	-	2/16
Cardiac event	-	+	-	-	+	-	-	-	-	-	-	-	-	-	-	-	2/16

*,**,*** and **** refer to 4 different families. Cases without asterisk are from different families

**Table 2:** Amino-acids values at diagnosis

	Age (years)	Patient values at diagnosis						Normal range adapted with age					
		Plasma (μmol/l)			Urine (μmol/mmol creatinine)			Plasma (μmol/l)			Urine (μmol/mmol creatinine)		
		Lys	Arg	Orn	Lys	Arg	Orn	Lys	Arg	Orn	Lys	Arg	Orn
1 *	1.5	116	82	47	3664	305	230	89-241	34-106	24-92	16-69	<8	<8
2 *	0.8	78	82	47	NA	NA	NA	96-262	41-117	22-114	13-79	<11	<8
3 **	16.2	34	14	9	32	7	2	120-268	40-124	25-125	7-58	<5	<5
5 ***	2.2	52	13	15	822	36	16	85-241	38-110	23-91	10-46	<9	<7
6	1.3	35	9	5	1372	107	31	89-241	34-106	24-92	16-69	<8	<8
8 ****	0.7	113	20	16	2990	195	67	96-262	41-117	22-114	13-79	<11	<8
9	2.2	74	17	8	1849	188	125	85-241	38-110	23-91	10-46	<9	<7
10	1.3	121	28	16	1721	231	135	89-241	34-106	24-92	16-69	<8	<8
11	12.6	33	7	10	33	7	10	141-224	35-115	20-132	10-56	<6	<6
12	3.4	77	27	11	886	155	38	85-241	38-110	23-91	10-46	<9	<7
13 ****	10.3	38	16	21	747	398	95	141-224	35-115	20-132	10-56	<6	<6
14 *	0.1	206	23	11	1504	121	58	106-246	35-91	22-154	22-171	<14	<19
15 *	0.005	177	33	69	673	129	75	106-246	35-91	22-154	22-171	<14	<19
16 ***	0.008	137	19	13	927	68	32	106-246	35-91	22-154	22-171	<14	<19

*,**,*** and **** refer to 4 different families. Cases without asterisk are from different families

### Patients – Circumstances of diagnosis – Death

Sixteen patients with LPI (9 families, 12 males, 4 females), followed at the Necker Children’s Hospital from 1977 to 2015, were enrolled in the study. Five different geographical origins were found: Maghreb (*n* = 4), France (*n* = 2), Turkey (*n* = 1), Lebanon (*n* = 1), and Guinea (*n* = 1). A consanguineous background was known for 12/16 patients. Uneventful pregnancy, spontaneous delivery, and normal birth parameters were noted in most cases (mean weight 3021 g (SD: 397 g), mean height 49.4 cm (SD: 1.5 cm)). One girl was born preterm due to premature labour (34^th^ week of amenorrhea). Another underwent a caesarean due to breech presentation.

The mean age at LPI diagnosis was 4.1 years (SD: 5.3 years). Patients were followed up for a mean of 11.4 years (SD: 10.4 years).

Symptoms at presentation were failure to thrive in 9/16 patients (2 patients with chronic diarrhoea, 3 with vomiting and anorexia associated with aversion to protein-rich food) and hyperammonemia in 10/16 patients who were symptomatic, including hypotonia in 3 and coma in 2 patients, thrombopenia in 8/16 patients, anaemia in 7/16 patients, and hepatosplenomegaly in 10/16 patients. Developmental disability had already been noticed in 5 patients at diagnosis (mean age 7.0 years; range 1.5 – 16.1 years). Five patients were diagnosed within the context of familial screening with only few symptoms that were limited to hepatosplenomegaly and/or vomiting. One patient presented with proteinuria up to 1 g/d.

Six patients (2 females and 4 males) died at a mean age of 4.0 y (SD: 3.2 years). Whereas initial symptoms were similar in patients from the same family, the evolution of symptoms and the prognosis were completely different in terms of organ involvement and death within the same family (Table 1).

### Pulmonary involvement

During follow-up, infiltrative lung disease (ILD) was reported in 10/16 patients. The pulmonary characteristics of the patients have recently been published [8]. All 10 patients had pulmonary alveolar proteinosis (PAP) confirmed by BAL, biopsy or autopsy. Five patients also developed lung fibrosis independently from the severity of PAP. One patient had associated alveolar haemorrhage within the context of thrombopenia. BAL was negative in two cases, although biopsies confirmed the diagnosis. When performed, HRCT scan showed patterns of ILD (7/7). Respiratory symptoms were never the initial presentation of LPI. Pulmonary involvement was initially limited to pathological imagery without symptoms in 4 patients (mean age at diagnosis 5.5 years). Although two of them developed remittent dyspnoea with moderate limitation of activity, these 4 patients are the only patients living, despite lung involvement (actual mean age 29.6 years). The remaining 6 patients presented with earlier pulmonary disease (mean age at diagnosis 2.24 years). They all died from respiratory failure at a mean age of 4.0 years (SD: 1.5 years) (Table 1). The age of PAP onset did not differ significantly between the patients who died and those who lived. The onset of an acute respiratory failure was always associated with a viral or bacterial pulmonary infection, and always led to death in the short- or middle-term. PAP was significantly associated with death (*p* = 0.034).

### Renal disease

Renal tubulopathy was observed in all screened patients (11/11). During the follow-up, 7 patients developed a chronic glomerular kidney disease.

Five of them have recently been reported including one 13.8-year-old patient with a membrano-proliferative glomerulonephritis [9]. In this patient, the presence of immune complexes associated with C1q and C3 deposits within the sub-endothelial space and the glomerular basal membrane, a positive Farr test and low complement CH50 and C4 levels first led to a diagnosis of lupus-like nephritis treated with corticosteroids before an atypical evolution with monoclonal IgG1 kappa deposits on kidney biopsy. Finally, this patient partially recovered, with persistent proteinuria (approximately 1 g/d.) after treatment with corticosteroids, mycophenolate mofetil and angiotensin-converting-enzyme (ACE) blocker.

**Fig. 2:**
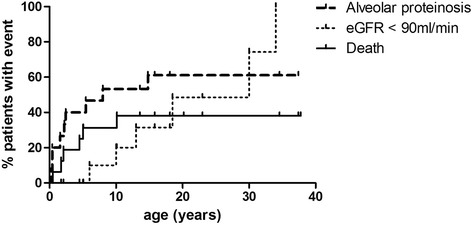
Death, renal disease and alveolar proteinosis observed during LPI. (percentage patient with event)

Six other patients developed chronic kidney failure at a mean age of 18.6 years (SD: 10.3 years). The mean follow-up time from the onset of kidney failure was 8.4 years (SD: 9.4 years). Changes in the eGFR were heterogeneous and were not associated with the duration of kidney disease. To date, GFR has remained above 60 ml/min in one patient (20 years), between 30-59 ml/min in 3 patients (13.6, 18.1 and 37.8 years), between 15-29 ml/min in 2 patients (34.6 and 37.8 years). Histological findings were also heterogeneous with amyloid lesions in one patient with a GFR estimated at 30 ml/min. Medullar fibrosis associated with histiocytic and lymphoplasmocytic inflammatory infiltrates in the interstitium were described in a newly diagnosed girl with an eGFR of 45 ml/min who is now receiving immunosuppressive therapy. At the age of 20 years, 50% of the patients in the cohort had GFR < 90 ml/min (Fig. 2). Microalbuminuria followed by significant glomerular proteinuria was observed in 5 patients, reaching up to 3 g/d. in 3 patients with lupus-like nephritis, amyloid deposits and unknown histology.

### Haematological disorders and autoimmunity

Five patients met sufficient criteria for a diagnosis of hemophagocytic-lymphohistiocytosis (HLH). Bone marrow aspirates were obtained in only 6 patients. Nevertheless, anaemia (Hb < 10 g/dl) and thrombopenia (<100 G/l) were reported in 7 and 5 patients, respectively. Hepatosplenomegaly was observed in all patients. Ferritinemia and triglyceridemia fluctuated, but pathologic concentrations were found in 11/12 (>500 μg/l) and 10/12 patients (>3.0 mmol/L), respectively. Three patients presented with hypofibrinogenaemia <1.5 g/L. Other signs were observed, including transaminase abnormalities, hypoHDL-cholesterol, and elevated lactate dehydrogenase (LDH). A specific treatment for HLH was required for 2 patients. Both received corticosteroids and cyclosporine, which was efficacious in only one. Two other patients required platelet transfusions.

Autoimmunity, specifically lupus nephritis, vitiligo and immune thrombocytopenic purpura, was reported in 3/16 patients.

### Digestive and nutritional concerns

An aversion to protein-rich food was noticed in 9/13 patients with associated diarrhoea and vomiting leading to episodic use of enteral nutrition for 4 patients and parenteral nutrition for 3. All patients (16/16) presented with failure to thrive at a mean age of 2.0 years (SD: 3.6 years) at diagnosis. Growth-hormone improved the height of 2/2 patients who presented with a concomitant deficiency. Among the 11 patients with available data, the available mean last height reached -2.51 SD (SD: 2.31). Osteopenia was reported in 12/15 patients of whom 3 developed fractures. Finally, multiple other deficiencies were also observed, such as hypocarnitinaemia (in 3 of the 4 screened) and low plasma levels of selenium (in 4 of the 5 screened).

Two patients experienced recurrent acute pancreatitis. Triglyceridemia varied up to10.3 mmol/L, but remained moderate in one patient during a crisis (5 mmol/L). In this patient, an abdominal CT scan, MRI and endoscopic ultrasound of the biliary tract and pancreas excluded gallstones and did not find any evidence of autoimmune reaction. No other cause was found. There was no history of alcohol consumption, the calcium and immunoglobulin G type 4 levels were normal, and there was no mutation in the cystic fibrosis transmembrane conductance regulator (*CFTR*), serine protease inhibitor kazal 1 (*SPINK1*) or cationic trypsinogen (*PRSS1*). Statin administration in one patient and taking the maximal dose of fenofibrate or gemfibrozil in the other patient did not prevent further episodes of pancreatitis.

### Cerebral impairment

Five among 16 patients presented with acute hyperammonemic encephalopathy with seizures in 4 and coma in 3 patients. Persistent hypotonia or developmental disability was recorded for 8/14 patients without a significant association with elevated glutamine. Another patient presented with seizures due to complicated cholesteatoma.

### Cardiovascular involvement

Two severe cardiac events occurred, in two distinct patients: i) an acute inferior and inferolateral myocardial infarction associated with diffuse coronaropathy treated with angioplasty and active stents in the intraventricular coronary artery, in a 34-year-old non-smoking man who concomitantly presented with mild kidney failure and hypertension and benefited from treatment with an ACE inhibitor; ii) a sinus arrest revealed by fatigue and malaise in a 22-year-old patient, that required a pace-maker. The patient had no personal or familial history of cardiac disease, hyperkalaemia or toxic ingestion. Cardiac amyloidosis has not specifically been looked for.

### Biochemical results (Table 2)

**Fig. 3:**
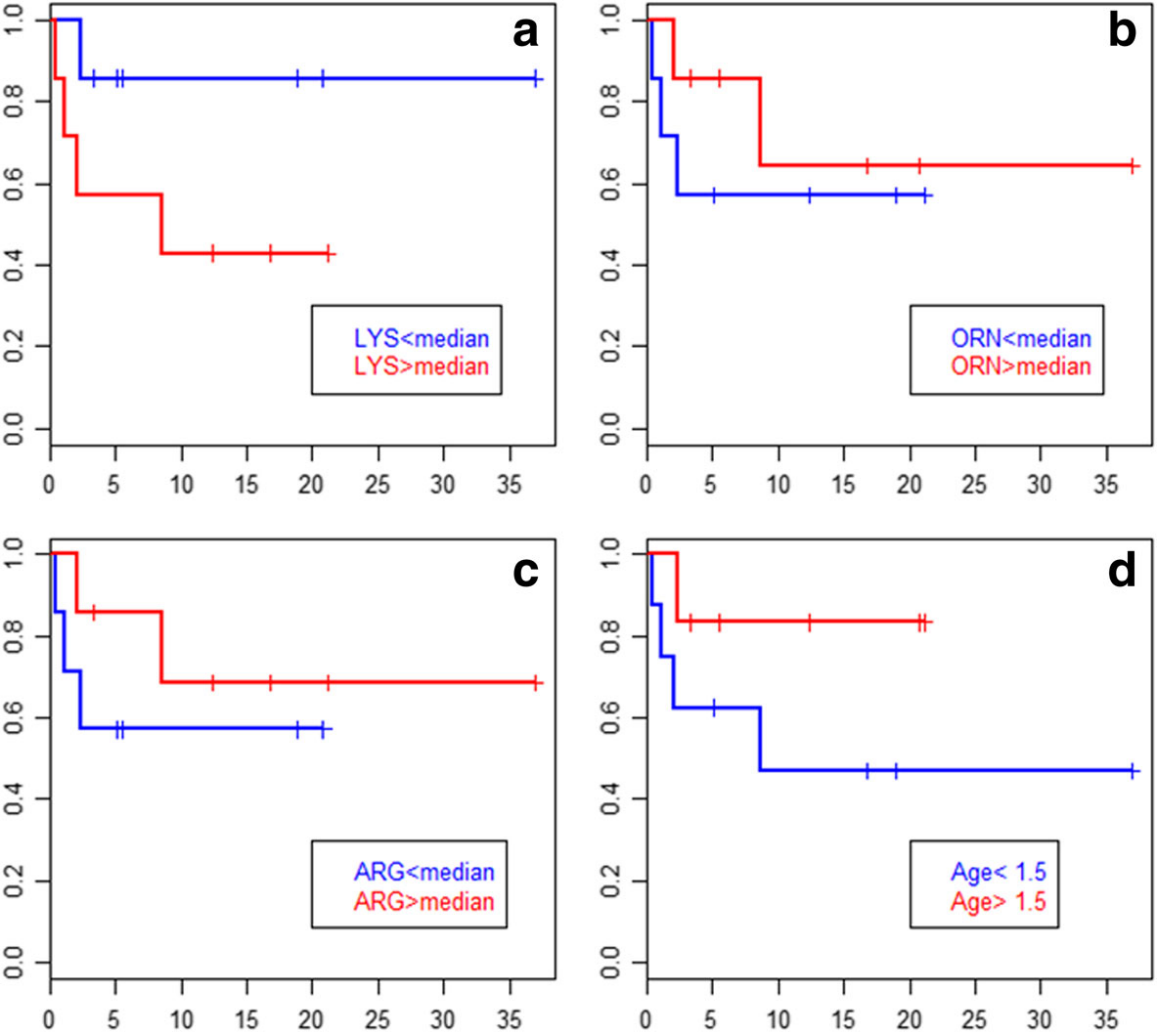
Kaplan-Meier overall survival plots for LPI patients. (red vs blue lines: patients with values above vs below a cutoff, resp., horizontal axis unit : year). Panels **a**-**c**: patients grouped by plasma dibasic amino-acid levels above or below the patients’ median at diagnosis. Panel **d**: patients grouped by age at diagnosis greater or lesser than 1.5 years. Increased age-adjusted plasma lysine and earlier age at diagnosis were borderline predictors of poorer outcome (see text)

Data at diagnosis were available for 14 patients. Biochemical results at diagnosis were unavailable for 2 patients whose diagnosis was not made in our centre. The mean plasma concentrations of lysine, arginine, ornithine and glutamine were 90 μmol/L (SD: 53, range: 33-206), 28 μmol/L (SD: 23, range 7-82), 22 μmol/L (SD: 18, range 5-69) and 978 μmol/L (SD: 317, range: 518-1507), respectively. These results are consistent with the literature [10]. In urine, the mean concentrations of lysine, arginine and ornithine were 1324 μmol/mmol creatinine (SD: 1057, range: 32-3663), 150 μmol/mmol creatinine (SD: 115, range: 7-398) and 70 μmol/mmol creatinine (SD: 64, range: 2-230), respectively. Of note, urinary levels of dibasic amino-acids were close to normal at presentation in some cases (age 16.2 and 12.6y.), and increased levels were found only on repeating the analyses because of high clinical suspicion. There was a trend towards higher plasma and urinary levels of cationic amino-acids at diagnosis in patients who subsequently died compared to living patients (Wilcoxon, *p* = 0.08; logrank, *p* = 0.17, Fig. 3). In addition, earlier age at diagnosis was a borderline predictor of shorter overall survival (logrank, *p* = 0.16; Fig. 3). Bivariate prediction of survival by age-adjusted plasma lysine levels and age at diagnosis suggests that their influence on prognosis is independent and additive (logrank, *p* = 0.10). Patients with PAP had a non-significant trend towards higher plasma lysine levels than those without PAP (Mann-Whitney, *p* = 0.11)

### Therapeutic management

All the patients received a hypoproteinemic diet adapted for their age from diagnosis to the last record. All were supplemented with citrulline (mean: 106.2 mg/kg/d.; SD: 46.2) and received vitamin supplementation. Three patients transiently received lysine supplementation, 4 arginine and 4 carnitine, with no difference in prognosis. Seven patients received ammonia scavengers, including sodium phenylbutyrate (*n* = 6) and/or sodium benzoate (*n* = 3) for hyperammonemia >80 μmol/L, without a difference in terms of prognosis. All patients with PAP underwent whole-BAL. Seven patients received corticosteroids, 3 within the context of respiratory failure and HLH with no improvement in respiratory function, 2 with isolated HLH, and 2 with kidney failure with an inflammatory pattern on biopsy. Three adult patients received ACE-inhibitors to control proteinuria.

### Molecular investigation

We characterised genotypes of 9 LPI patients from 6 non-related families (Table 3). Five different mutations in the *SLC7A7* gene were identified, including 3 point missense mutations (c.726G > A, c.753G > C and c.1417C > T) and two small deletions (c.254_255delTT and c.1185_1188delTTCT), which led to a frameshift and created a premature stop codon. Among these 5 mutations, two were recurrent in 2 families (c.726G > A and c.1185_1188delTTCT) [11, 12]. Only the c.753G > C was novel. The pathogenicity of this mutation was disputed based on predictive data and the existence of a previously reported mutation in the same codon (c.753G > T, p.Glu251Asp) [11]. Except for one patient who carried two mutations in a heterozygous state, the remaining patients with a known consanguineous link (5/6 families) carried one mutation in a homozygous state. Seven patients in the cohort did not have a molecular analysis due to the absence of a sample, but 4 in the same family were related to a least one genotyped proband. No obvious genotype-phenotype relationship was identified because the 6 patients who carried the same genotype did not have the same prognosis and organ involvement.

**Table 3:** Mutations in *SLC7A7* gene

Family	1	2	3	4	5	6
Tested patient(s)	2, 14, 15	3, 4	16	8	10	12
Non tested related patients	1		5, 7	13		
consanguinity	+		+	+	+	+
Mutation(s)	c.726G > A(p.Trp242*)	c.753G > C (p.Glu251Asp)	c.1185_1188deITTCT	c.726G > A(p.Trp242*)	c.254_255deITT	c.1417C > T (p.Arg473*)
		c.1185 1188deITTCT^a^				
Publication(s)	Sperandeo et al, 2000	Mykkanen et al, 2000^a^	Mykkanen et al, 2000	Sperandeo et al, 2000	Mykkanen et al, 2000	Mykkanen et al, 2000

## Discussion

Four systems have been described for cationic amino-acids transport through plasma membrane in mammals: y^+^, b^0,+^, B^0,+^ and y^+^L. These systems differ in terms of specificity for amino-acids, tissue localisation and dependency on sodium [10]. The y^+^LAT-1 and 4F2hc subunits heterodimerise to form the part of the y^+^L system that is responsible for the exchange of cationic amino-acids for neutral amino-acids plus sodium at the basolateral plasma membrane of polarised cells, such as epithelial cells of the proximal renal tubules, small bowel, lung and leukocytes. Only mutations in the *SLC7A7* gene encoding the y^+^LAT-1 subunit have been identified in LPI patients, but no mutation has been reported in *SLC3A2* encoding 4F2hc. Interestingly, cystinuria (OMIM 220100) is also caused by defects in cationic amino-acids transport in the b^0,+^system [13–15].

Initially described by Perheetupa and Visakorpi in 1965, LPI has been reported worldwide [15]. There is a higher prevalence in Finland reaching 1/60 000. However, LPI has also been documented in Japan, Turkey, Italy and Maghreb [3, 4, 12, 16]. As expected with an autosomal recessive disease, inbreeding was important in our cohort.

Since the first description of LPI, a great deal of clinical heterogeneity has been observed among patients. LPI is often revealed by the appearance of chronic digestive symptoms and failure to thrive after presumptive diagnoses of coeliac disease, other causes of malabsorption or “food allergies” [3, 4]. However, the onset can also be sudden with hyperammonemic encephalopathy. All but one patient had hepatosplenomegaly at diagnosis, a feature that should alert practitioners to the possibility of LPI.

As observed in our cohort, patients from the same family can have entirely different evolution of symptoms, which may hinder the ability to provide a definitive prognosis (Table 1). Inter-familial clinical heterogeneity (alveolar proteinosis, cerebral impairment, cardiac event) in patients displaying the same genotype (families 1 and 4, c.726G > A homozygous) is also common. To date, 51 mutations in *SLC7A7* have been reported [13–15]. Here, we report 5 different mutations, 4 of which were previously described and 1 was a novel mutation. With the lack of a clear genotype-phenotype relationship, the identification of mutations in patients may permit confirmation of the diagnosis but is not useful in predicting the severity of the clinical course [16]. In a Finnish transcriptomic study on 13 patients, the expression of 926 genes differed significantly from controls, involving basic cellular functions such as cell cycle, signalling, ion transport or apoptosis. The extent of transcriptional effects has been hypothesised to explain the variety of symptoms observed in LPI [13]. Although 12/16 of our patients were males, this overrepresentation was not statistically significant (Fisher’s exact test) and has not been described in other cohorts [17, 18].

Pulmonary involvement appears to play a major role in the prognosis of the disease. The characteristics of the respiratory disease in our cohort were recently published [8] and consist clinically, radiologically and histologically of an early and persistent PAP, which, in some cases, can be associated with fibrosis. The respiratory complications have no obvious association with the clinical or radiologic severity of the PAP. Radiological findings usually show an interstitial or alveolo-interstitial pattern with intralobular lines, interlobular septa thickening and frequent cysts and ground glass opacities. Fibrosis may develop independently from PAP. The early onset of pulmonary disease is associated with a severe prognosis as previously described [18]. There were more asymptomatic patients in the Finnish study (65%), in which chest X-ray was used to diagnose the disease [18]. Santamaria et al. observed that 8/9 patients were asymptomatic despite the presence of infiltrative lung disease on the HRCT-scan [17]. To date, the HRCT-scan is recommended to detect lung involvement [8].

PAP is a result of defects in the surfactant homeostasis composed of proteins and phospholipids that are synthesised by type 2 pulmonary epithelial cells (PECs). PECs are also responsible for the recycling of 70 to 80% of the surfactant, whereas the rest is phagocyted by alveolar macrophages (therafter, AM) or caught in the lymphatic circulation. PAP can be either acquired or inherited. In children, genetic causes of PAP include LPI, methionyl-tRNA synthetase (*MARS*), and mutations in *Granulocyte-macrophage colony stimulating factor* (GM-CSF) receptors genes *CSF2RA* and *CSF2RB* [19]. In adults, the most frequent form of PAP is autoimmune and mediated by anti-GM-CSF antibodies. In this latter form, GM-CSF injections have been shown to be effective [20]. GM-CSF is essential for the differentiation of AMs by enhancing their ability to uptake and catabolise remaining surfactant components. Although *SLC7A7* gene expression is enhanced by GM-CSF, AM differentiation is not impaired in LPI patients [21], and GM-CSF appears to be inefficient or even dangerous in hemophagocytic-lymphohistiocytosis (HLH) related to LPI.

In alveolar macrophages (AMs), y + LAT-1/4F2hc is responsible for the influx of arginine [22]. y + LAT-1/4F2hc defects lead to a decrease of intracellular arginine, which is the main substrate for nitric oxide synthase (NOS), the enzyme responsible for the production of nitric oxide (NO) [23]. NO plays a critical role in the lytic processes of macrophages as well as in vasoregulation, platelet aggregation and neurotransmission [24]. The actual paradigm for PAP-LPI is that AMs present a defect in phagocytic function that impairs the homeostasis of surfactant [25]. Moreover, LPI-related PAP is clearly aggravated by infections. All of the children who died did so from bacterial or viral pneumonias that complicated PAP. It has been recently described that the Toll Like Receptor 9 (TLR 9) pathway, which is essential for bacterial and viral defence, is impaired in macrophages of patients with LPI, with decreased interferon type I production [23]. Intracellular NO accumulation secondary to defects in arginine transport has been proposed to explain the pro-inflammatory pattern in LPI, but Kurko recently showed that the NO levels were on the contrary decreased in patients with LPI [3, 4, 23]. In addition, the pro-inflammatory pattern could result from the upregulation of TLR2/1 and TLR4 pathways leading to higher levels of tumor necrosis factor alpha (TNF alpha) and interleukin-12 [23]. The role of corticosteroids also remains controversial. There is no pathophysiological rational or clear benefit that justifies promoting this treatment, which is associated with an increased risk of infections. A prompt antibiotic regimen should therefore be initiated after microbiological documentation of an infection. Vaccination and physiotherapy for airway clearance are also important in the management of this chronic respiratory complication.

Interestingly, we were surprised to find some evidence for a higher plasma concentration of lysine in patients with a poor prognosis. The role of lysine or cationic amino-acids in PAP has not clearly been determined. Because the intra-alveolar cationic amino-acid levels and the possible role of pulmonary epithelial cells are unknown, further elucidation of their roles has been hampered. Of note, a 13-year-old boy died 26 months after a heart-lung transplantation for LPI-AP and recurrent PAP on the graft, which suggests that macrophages play a major role in the pathophysiology of the disease [26]. It was recently reported that the defect of SLC7A7/y^+^LAT-1 leads to the increased activity of another cationic amino-acid transporter such as SLC7A1/CAT1, as suggested by Tringham and al., who used a transcriptomic approach [13]. Nevertheless, this transporter is expressed in the basolateral plasma membrane of epithelial cells, but apparently not in AMs [22, 23, 27]. We speculate that the lack of SLC7A1 in AMs may increase susceptibility to lysine depletion in this cell type thereby possibly leading to both higher risk of alveolar proteinosis and moderate increase of lysine levels in plasma of patients with poorer outcome. Nevertheless, other factors would be involved to explain why plasma arginine and ornithine are not similarly involved. Alternatively, the association between moderate increases in lysine levels and poorer outcome might reflect putative downstream regulatory mechanisms, for instance a need to more strongly repress lysine oxydation in order to maintain protein synthesis. Such a putative mechanism would lead to increased lysine during catabolic stresses.

Except for the presence of early and persistent tubular involvement, renal disease primarily affects older patients (mean age of onset 17.7 years; SD 9.73 years). When kidney dysfunction is suspected, GFR should be actually measured rather than just estimated [28]. Glomerular involvement with kidney failure was reported in half of our patients after the age of 20. Despite a common genotype, the 3 available renal biopsies showed different histological patterns, such as glomerular amyloidosis, interstitial fibrosis or mesangial sclerosis, consistent with a previous report by Tanner, who evaluated a Finnish cohort [28]. The mechanisms remain obscure. Like in our patient with lupus nephritis, some autoimmune nephropathies associated to LPI have benefited from classical immunosuppressive therapy [9, 29]. Proximal tubulopathy seems to be an early hallmark of the disease as it was observed in all of our patients. Fanconi syndrome has also been reported, though the underlying mechanism remains hypothetical [30]. An elevation of intracellular lysine concentrations in the tubule would increase tubular permeability and enhance apoptosis through NADPH oxidase [31]. Therapeutic concerns focus on the early introduction of ACE-blockers to diminish proteinuria and possible hypertension [28]. Interestingly, 3 patients have successfully benefited from renal transplantation, as reported in the literature [28].

Various inflammatory and auto-immune manifestations including vasculitis can complicate LPI [32]. Interestingly, defects of phagocytosis have been observed in circulating LPI-associated monocytes and non-alveolar macrophages, as described in auto-immune diseases [25, 33]. Moreover, classical immunosuppressive treatment improves LPI inflammatory complications [32]. Whereas the T-cell response is normal, the humoral immune response is defective in some patients with LPI and an increased risk of infection [34]. However, we observed only one case of severe sepsis in our cohort.

HLH is another frequent immune complication observed in LPI [3, 4]. Primary or secondary HLH is due to an uncontrolled and self-sustained hyper-inflammatory response involving NK-cells and cytotoxic T-lymphocytes that leads to the uncontrolled activation of macrophages and pro-inflammatory cytokine secretion [35]. Such cytokine secretion has been reported in 4 patients with LPI with notably high levels of soluble CD25 [36]. In our cohort, haemophagocytosis was histologically documented in only 6 patients through bone marrow aspiration. Most of the time, HLH symptoms were limited to characteristic splenomegaly, hypertriglyceridemia, elevations in ferritin levels and low fibrinogen, none of which lead to further investigation because they were not life-threatening. Nevertheless, hypertriglyceridemia was associated with recurrent acute pancreatitis in 2 patients who did not respond to treatment with statins, fibrates or gemfibrozil. Hypertriglyceridemia is considered a risk for pancreatitis when the levels are >1000 mg/dL (11.2 mmol/L) [37], but the question arises of whether moderate levels can induce pancreatitis in such patients. Interestingly, an association between bone marrow abnormalities suggesting HLH and pancreatitis in LPI patients has already been described [38], underlining this hypothesis. Nevertheless the existence of predisposing factors for hypertriglyceridemia such as Asn9 variant of lipoprotein lipase discovered in a brotherhood among patients should remind the probable role for other genes in the pathogenicity of the different symptoms observed in the disease.

Failure to thrive is a hallmark of LPI and is associated with protein depletion intrinsically associated to LPI but also secondary to malabsorption. Growth hormone supplementation was effective in patients with associated growth hormone insufficiency as already reported [39]. Its use has not been evaluated yet in LPI. We did not find any obvious benefit from oral supplementation with citrulline, lysine or arginine. Although criteria for osteopenia were limited to bone radiography, osteopenia was reported in the majority of the patients. Parto et al. also described histologic signs of osteoporosis in 8/9 patients. Collagen synthesis in skin fibroblasts was decreased. The mechanism involved in the development of osteopenia seems to be more associated with synthesis defects secondary to protein depletion than increase of degradation by osteoclasts or inflammation [40]. Finally, multiple nutritional deficiencies are observed in patients with LPI in the area of global nutritional depletion. Selenium deficiency can lead to myocardiopathy and myopathy [41]. Targeted nutritional monitoring is therefore essential.

A secondary urea cycle disorder with hyperammonemia has been well described in LPI. Indeed, low arginine and ornithine is thought to primarily lead to the functional depletion of the urea cycle intermediates [10]. More than half of the patients presented with cognitive disorders. Though this complication has not been reported in the literature, it can be easily explained by chronic hyperammonemia. With regard to chronic dyslipidaemia, we did not observe any cerebral strokes, though an unexpected myocardium infarction did occur. This latter was definitively associated with diffuse coronaropathy. We are the first to report a cardiac involvement but another vascular disease, moyamoya vasculopathy, was recently described in LPI [42]. The cycle of low plasma levels of arginine resulting in NO depletion, decreased arterial and coronary vasodilatation and ultimately ischemia has already been described [43]. Cardiovascular monitoring is thus necessary in young adults with LPI who present hypertriglyceridemia, recurrent kidney failure and other risk factors for arterial disease. Moreover, we also report a conduction disorder whose mechanism is obscure. Based on renal and hepatic biopsies from 2 different patients, we noted that amyloidosis can complicate LPI. Whether amyloid lesions were responsible for the conduction block is unclear because echocardiography was not as suggestive. Nonetheless, this potential correlation urges caution.

General therapeutic management remains empirical with 3 major axes: prevention of hyperammonemia, nutritional supplementation and prevention of specific complications. There is a general consensus that a hypoproteinemic regimen should be initiated with an objective of 1 g/kg/d. associated with L-citrulline supplementation, L-carnitine 20-50 mg/kg/d., vitamins and other nutritional supplementation, if necessary [3, 4]. Ammonia scavengers such as sodium benzoate or phenylbutyrate are used based on glutamine levels. L-citrulline is used to correct the intracellular defect of arginine through arginosuccinate-synthase and argininosuccinate-lyase. Because arginine was initially thought to be increased in macrophages, the dosage of L-citrulline dosage was a subject of controversy because of the possibility that it could increase inflammatory damages through its conversion to arginine and NO. L-Citrulline supplementation is thus usually limited to 100 mg/kg/d [3, 4]. Nevertheless, it was shown that arginine and NO were actually lacking in macrophages and that low plasma levels of arginine may be associated with cardiovascular disease. Therefore, the dose may need to be reassessed. Also, an alimentation restricted into long chain fatty acids and supplemented with medium chain fatty acids is questionable, in order to decrease the levels of triglycerides and prevent the risk of pancreatitis. Our data show a borderline significant association between relatively increased lysine levels (within normal to low values) and poor prognosis, calling for new investigations on the mechanisms of lysine transport and metabolism in LPI patients. Finally, this study reveals the importance of careful renal and cardiovascular monitoring. ACE-blockers and hypolipidemic agents may play an essential role in the therapeutic management and prevention of several complications.

## Conclusions

This study presents a general review of LPI symptoms and raises new questions on prognostic factors and the evolution of the disease in young adults. PAP is an early complication that affects the general prognosis, whereas kidney failure develops primarily in older patients. Young adults would benefit from systematic cardiovascular monitoring and general recommendations with regard to management of dyslipidaemia and nutritional supplementations. Moreover, autoimmune complications should systematically be discussed in the presence of atypical symptoms as they appear accessible to classical therapeutics. High plasma lysine levels show some association with poor prognosis yet the mechanism of pathology is unclear warranting additional investigations in different patient cohorts and by in vitro assays.

## Abbreviations

ACE: Angiotensin-converting-enzyme
AM: Alveolar macrophage
BAL: Broncho-alveolar lavage
GFR: Glomerular filtration rate
GM-CSF: Granulocyte-macrophage colony stimulating factor
HLH: Hemophagocytic lymphohistiocytosis
HRCT: High resolution computed tomography
ILD: Infiltrative lung disease
LDH: Lactate dehydrogenase
LPI: Lysinuric protein intolerance
NK: Natural Killer
NO: Nitric oxide
NOS: Nitric oxide synthase
PAP: Pulmonary alveolar proteinosis
PCR: Polymerase chain reaction
SD: Standard deviation
